# Evolution and spread of Xanthomonas citri subsp. citri in the São Paulo, Brazil, citrus belt inferred from 758 novel genomes

**DOI:** 10.1099/mgen.0.001338

**Published:** 2025-01-16

**Authors:** Caio Felipe Cavicchia Zamunér, Dennis Carhuaricra-Huaman, Roobinidevi Ragupathy, James Redfern, Carmen L. Rodriguez-Cueva, Franklin Behlau, Mark C. Enright, Henrique Ferreira, João C. Setubal

**Affiliations:** 1 Departamento de Biologia, Instituto de Biociências, Universidade Estadual Paulista, Av. 24A, 1515, Bela Vista, Rio Claro, 13506-900, São Paulo, Brazil; 2 Departamento de Bioquímica, Instituto de Química, Universidade de São Paulo, São Paulo, SP, Brazil; 3 Department of Life Sciences, Manchester Metropolitan University, Chester Street, Manchester, M1 5GD, UK; 4 Department of Natural Sciences, Manchester Metropolitan University, Chester Street, Manchester, M1 5GD, UK; 5 Fundo de Defesa da Citricultura - Fundecitrus, Av. Dr. Adhemar Pereira de Barros, 201, Araraquara, 14.807-040, São Paulo, Brazil

**Keywords:** citrus canker, evolution, genomes, São Paulo state citrus belt, *Xanthomonas citri*

## Abstract

The São Paulo state citrus belt in Brazil is a major citrus production region. Since at least 1957, citrus plantations in this region have been affected by citrus canker, an economically damaging disease caused by *Xanthomonas citri* subsp. *citri* (*Xcc*). For about 50 years, until 2017, a citrus canker eradication programme was carried out in this region. In this work, our aim was to investigate the effects of the eradication programme on genetic variability and evolution of *Xcc*. To this end, we sequenced and analysed 758 *Xcc* genomes sampled in the São Paulo citrus belt, together with 730 publicly available *Xcc* genomes from around the world. Our phylogenomic analyses show that these genomes can be grouped into seven major lineages and that in São Paulo, lineage L7 is dominant. Our time estimate for its appearance closely matches the date when citrus production expanded. L7 can be subdivided into lineages L7.1 and L7.2. In our samples, L7.2, which we estimate to have emerged around 1964, is by far the most abundant, showing that the eradication programme had little impact on strain diversification. On the other hand, oscillations in the estimated effective population size of L7.2 strains over time closely match the shifts in the eradication programme. In sum, we present a detailed view of the genomic diversity of *Xcc* in the world and in São Paulo, the largest such effort in terms of a number of genomes for a crop pathogen undertaken so far. The methods employed here can form the basis for active genomic surveillance of *Xcc* in major citrus production areas.

**Impact Statement**

Citrus canker is a disease that impacts all major citrus-producing areas around the world, causing considerable economic losses. Since 2002, many genomes of *Xanthomonas citri* subsp. *citri* (*Xcc*), the causative agent of citrus canker, have been sequenced and analysed, and the corresponding studies have increased our knowledge of the genomic basis of citrus canker. Newer genomic analysis techniques coupled with a sufficient number of genomes for a given pathogen sampled over time now allow for a more detailed view of the evolution and spread of the pathogen, thus contributing to the understanding of citrus canker outbreaks and dissemination. In this work, we have sequenced 758 novel *Xcc* genomes (thus doubling the number of public genomes for this pathogen) and have applied the referred techniques to gain a deeper understanding of the evolution and spread of citrus canker around the world and in the São Paulo, Brazil, citrus belt. The main results are that we have identified seven major lineages of the pathogen and their geographic occurrence, and we now know which lineages are present in the São Paulo citrus belt and how they have evolved over space and time. The techniques employed and the results achieved can form the basis of future citrus canker management actions in São Paulo as well as in other citrus-producing regions.

## Data Availability

The 758 XccA genome sequences generated for this study are available in GenBank (National Center for Biotechnology Information), with BioProject and BioSample accession numbers given in Table S3.

## Introduction

Citrus canker is a disease caused by the Gram-negative bacterium *Xanthomonas citri* subsp. *citri* (*Xcc*), which can infect all commercially important citrus cultivars wherever citrus plants are grown [1,2]. The typical symptoms of citrus canker are erumpent circular patches that evolve to become corky-like lesions that appear in all newly growing aerial parts of the plant [3]. Moreover, infected plants cannot be healed, and, in severe cases, disease will cause defoliation and premature fruit drop that greatly impact the productivity [1]. The spread of bacteria can be rapid, occurring mainly through the action of wind and rain in warm and humid climates. Control measures are either based on quarantine and exclusion programmes, in order to avoid the introduction and spread of the disease to canker-free areas, or on disease management in endemic areas. The latter, currently in use in the state of São Paulo (SP), the largest citrus-producing area in Brazil, is established by using windbreaks, planting less susceptible cultivars of citrus, controlling the citrus leaf miner and spraying copper bactericides [1,4, 5].

The introduction of citrus canker in Brazil likely occurred a few years before its first detection, from contaminated plants imported from Asia [6]. Originally identified in India around 1830 [7], the disease was first detected in Brazil in the Presidente Prudente municipality, in SP state, in 1957 [8]. As soon as it was identified in SP, the pathogen was also detected in neighbouring states, which planted infected nursery trees produced in SP to establish new orchards [9]. Along the years, several enforcement protocols were adopted by the citrus canker eradication programme (CCEP) to contain the spread of the disease. Among them, the eradication programme, applied in the state of SP between the years of 1999 and 2009, was one of the most successful and restrictive protocols, which kept the incidence of the disease at very low levels (below 0.2%) [5,9, 10]. From 2010, combined actions from different sectors of the society led to the end of the CCEP, which culminated in the current scenario of citrus canker endemism in the SP citrus belt. Currently, control of citrus canker in SP and several other states in the country is done by risk mitigation based on control of the disease in the field and decontamination of fruit in packing houses [5].

Several studies used genomic approaches to explore the evolutionary history of *Xcc* lineage diversification in relation to host range, as well as its spread in other countries [11,14]. These studies are essential for a better understanding of the evolution and spread of this phytopathogen, providing valuable information for citrus canker management and surveillance. In a recent work [14], the authors sequenced the genomes of 343 *Xcc* strains sampled in Florida (USA) between 1997 and 2016 and identified 2 separate clusters in the region. They also showed the role of hurricanes in spreading *Xcc* through the state and the ongoing presence of strains once believed to have been eradicated in several orchards. Brazil, particularly SP state, offers a unique opportunity for epidemiological studies, in which the relatively low incidence level of citrus canker, maintained for decades until 2017 by the CCEP, may have offered fewer opportunities for bacterial genetic diversification.

Previous studies explored *Xcc* genetic diversity in Brazil using different molecular techniques. Jaciani *et al.* [15] analysed the isolates based on probes for type III effector protein genes, identifying 49 different haplotypes but with short genetic distances among them. Nevertheless, the authors found a large diversity of haplotypes in SP and suggested that populations present in SP and Paraná have the same genotypic origin. Furthermore, populations of *Xcc* in southern states of Brazil may have been formed or influenced by other introductions from neighbouring South American countries. The relationship between geographical origin and genomic similarity was also observed by Carvalho *et al.* [16], in strains sampled between 1989 and 2000 from Brazil and neighbouring countries, suggesting multiple introductions of the bacteria in South America. The authors also mentioned the high genetic similarity among strains from SP. Similarly, in a study by Gonçalvez-Zuliani *et al.* [17], small variations were found among isolates from different municipalities in the state of Paraná, which suggests a low degree of spatial differentiation between populations in that state. While these studies offer significant local insights into the genetic variation of *Xcc* in Brazil, numerous questions persist.

The current scenario of recent endemism of citrus canker in the SP citrus belt possibly allowed the introduction of different strains in the area and led to an increase in the genetic variability of the bacterial population. The present study aimed to understand the global population diversity and reconstruct the evolutionary history of *Xcc* in SP by sequencing several hundred *Xcc* genomes from recently sampled isolates in SP and using a population genomic approach. The main results are that we have identified seven major lineages of the pathogen and their geographic occurrence, and we now know which lineages are present in the SP citrus belt and how they have evolved over space and time.

## Methods

### Bacterial isolates and identification

Leaf samples were obtained during the period of 2018–2021 from 13 different municipalities located throughout the major citrus-producing regions of the SP citrus belt (Fig. S1, available in the online version of this article). The sampled area has ~57 330 km^2^, with an approximate north-south distance of 350 km and east-west of 280 km. Each sample consisted of 200 canker symptomatic leaves collected from trees randomly distributed in orchards, preferably after the rainy season (to optimize bacterial isolation from newly formed lesions). The host tree species in all cases except one was *Citrus sinensis*; the exception was the Marapoama municipality, where strains were isolated from *Citrus latifolia*. For bacterial isolation, canker lesions were excised from symptomatic leaves and the surface was disinfected using subsequent two washes with ethanol 70% (v/v) (2 min), one with sodium hypochlorite 2% (v/v) (2 min) and two washes with sterilized distilled water. After disinfection, 10 µl of sterile distilled water was poured onto lesions and incubated at room temperature for 10 min, to exudate the bacteria from the mesophyll. Following incubation, sterile toothpicks were used to scrape around the canker lesions for subsequent inoculation in Nitrogen Yeast Glycerol medium (NYG)(5 g l^-1^ peptone, 3 g l^-1^ yeast extract and 20 g l^-1^ glycerol), supplemented with 16 µg ml^-1^ kasugamycin, 16 µg ml^-1^ cephalexin hydrate and 50 µg ml^-1^ cycloheximide, modified from Behlau *et al.* [18]. Plates were incubated at 29 °C for 96 h to allow bacterial growth. The identity of the *Xcc* candidates grown on plates was checked by colony PCR diagnosis according to Coletta-Filho *et al.* [19]. *Xcc* strain A306 (IBSBF 1594) DNA was used as positive control. All strains were stored in ultra-low temperature freezers at –80 °C in cryogenic tubes with NYG medium added with glycerol at a final concentration of 50% (v/v).

### Whole-genome sequencing, assembly and annotation

Genomic DNA libraries were prepared from pure culture material in DNA/RNA Shield buffer (Zymo Research, CA) using Nextera XT library prep kits (Illumina, San Diego, CA, USA). Genomic libraries were sequenced in MicrobesNG facility (University of Birmingham, UK) using a 250 bp paired-end protocol. Reads were adapter trimmed using Trimmomatic 0.30 [20] with a sliding window quality cutoff of Q_15_ [20], and species identity was confirmed using Kraken [21]. *De novo* assembly was performed with the program SPAdes v3.7 [22] with default parameters. Number of contigs (≥200 bp) in assemblies varied between 68 and 319, with N50 values between 105 308 and 343 324 bp and an average depth of coverage of 76× (range: 30× to 258×) (Table S1, Fig. S2).

Publicly available *Xcc* genomes and associated metadata were downloaded from GenBank. All assemblies used in our analyses had to pass the following quality control: genome size between 4 and 6 Mbp, fewer than 1000 contigs, at least 95% completeness and at most 5% contamination, as evaluated by CheckM [23]. Contigs with less than 200 bp were filtered out from assemblies. Table S2 shows public genomes that were excluded according to the quality criteria just described.

Our full dataset consists of 1488 *Xcc* genomes, including 758 novel genomes from Brazil and 730 publicly available genomes, many of which came from 3 recent studies [14,24, 25].

All 1488 genomes were annotated with Prokka v1.14 [26] with default parameters.

### Phylogenomic analysis of *Xcc*

Phylogenetic reconstruction of 1488 *Xcc* genomes was performed using Snippy v4.6.0 (https://github.com/tseemann/snippy) with isolate A306 (GenBank ID: NC_003919.1) as the reference. Recombinant regions were identified with Gubbins v3.3 [27] and removed from the alignment. Variant sites in the alignment were extracted using SNP-sites v2.5 [28]. The SNP alignment was used as input to IQ-TREE2 [29] for a maximum likelihood (ML) phylogenetic tree reconstruction under the general time reversible (GTR) substitution model +Γ and run for 1000 steps to obtain bootstrap values. The phylogeny of 841 Brazilian *Xcc* genomes was also constructed following the same steps. Trees were visualized and annotated using ggtree [30].

Population structure analysis of *Xcc* clade A strains (*n*=1488 genomes) was performed using fastBAPS [31] in R using the SNP alignment as input to identify major clusters. Pairwise SNP distance between all genomes was calculated using SNP-dists tool v0.8.2 (https://github.com/tseemann/snp-dists) from the SNP alignment.

### Pangenome reconstruction

The gff files of Prokka-annotated genomes were fed to Panaroo v1.2.7 [32] to identify core and accessory portions of the pangenome using the strict lean mode and default parameters. We performed a cluster analysis of the gene presence/absence matrix to identify genome groupings based on gene content using the Manhattan distance and Ward hierarchical clustering in pheatmap v1.0.12 package [33]. Principal component analysis (PCA) of the accessory portion was computed with function *prcomp()* in R v4.3 and visualized in RStudio using the ggplot2 package [34]. Chromosomal regions and plasmids were visualized using Easyfig [35] and BRIG [36]. We used GenBank-annotated reference plasmid sequences to compare plasmid gene content: pLJ207-7.3 (CP018853.1, strain LJ207-7) and the copper-resistance plasmid pCuR (CP018859.1, strain LH201).

### Time-calibrated phylogeny and phylogeographic reconstruction

A time-calibrated phylogeny was constructed on a subsample of 203 Brazilian genomes, including 72 historical genomes and 131 contemporary genomes with available collection dates. Before inferring the time-calibrated phylogeny, the existence of a temporal signal in the data was assessed with a regression analysis of the root-to-tip branch distances within the ML phylogeny, as a function of the year of collection, with TempEst v1.5 [37]. We used BEAST v1.10.4 [38] to date evolutionary events. To calibrate the molecular clock, we incorporated isolation dates for each genome (indicated by the year of collection). We employed a GTR+Γ substitution model and tested different combinations of the uncorrelated relaxed lognormal clock and tree priors (constant, exponential growth or Bayesian skyline demographic model). Each model was fitted using a Markov chain Monte Carlo (MCMC) run with 700 million iterations, sampling every 50 000 iterations. We evaluated the adequacy of sampling from the stationary distribution by ensuring that the effective sample size of key parameters exceeded 200 in Tracer v1.7.2 [39]. Additionally, we assessed the convergence by repeating the analyses and confirming that they reached the same stationary distribution. Since our alignments consisted solely of SNPs, we explicitly specified the number of constant nts in the model (https://groups.google.com/forum/#!topic/beast-users/QfBHMOqImFE). The maximum credibility tree was generated using TreeAnnotator v2.6.6 [38] and visualized with the ggtree package.

Lineage L7.2 was identified as a monophyletic group on the time-resolved phylogenetic tree. Genome sequences (*n*=168) from this lineage with associated geographical locations (latitude and longitude) were extracted to infer continuous phylogeography histories in BEAST v1.10.4 [38]. We selected the Cauchy relaxed random walk as a continuous trait model [40] and the same clock (uncorrelated relaxed lognormal) and demographic (Bayesian skyline) models described earlier. The MCMC was set to run for 500 million steps logging every 50 000 steps with a 10% burn-in. We used the R package Seraphim [41] to extract and map spatiotemporal information embedded in the posterior tree.

### Positive selection analysis

We assembled a codon alignment of each gene in the core-genome alignment of 203 Brazilian genomes using the A306 genome annotation. Signals for selection were inferred by applying FUBAR [42] implemented in HYPHY 2.2 software. FUBAR was run with 10 MCMC chains, each consisting of 1 000 000 iterations. Evidence of positive selection for each codon was identified at posterior probability with a value at least 0.90. Genes located in recombinant regions were removed from the analysis.

We assessed the impact of non-synonymous mutations in SodM (locus tag: XAC2386) stability using the protein stability prediction tool DDMut [43]. The SodM protein structure of *Xcc* has been determined (PDB:63EB [44]). This structure was used as a query for DDMut to determine the impact of missense mutations. DDMut predicts the changes in Gibbs free energy between WT and mutant protein structures. A positive value indicates a stabilizing or favourable mutation, while a negative value denotes a destabilizing or unfavourable mutation.

### Virulence gene characterization

Sequences of interest were identified using blastn against 65 effectors and 24 genes of the type III secretion system from Campos *et al.* [45]. For copper-resistance genes, we used reference genes *copL*, *copA*, *copM*, *copG*, *copC*, *copD* and *copF* found in plasmid pLH201.1 (GenBank accession CP018859.1) of the LH201 strain.

### Defence systems

We used the program DefenseFinder v1.3.0 [46] to locate anti-bacteriophage defence systems.

## Results

### Global diversity of *Xcc* pathotype A

We analysed a global collection of 1488 *Xcc* genomes, including 758 *Xcc* genomes from Brazil sequenced for the purpose of this study (Table S3). The *Xcc* tree indicates the existence of divergent clades representative of the well-known A, A* and A^w^ pathotypes (Fig. S3). The A pathotype was divided into three subclades (A1, A2 and A3), in agreement with results by Campos *et al.* [45], with the A1 lineage as the group with the most genomes (*n*=1465). This *Xcc* A1 dataset includes all genomes from Brazil (*n*=841), as well as from other South American countries (*n*=13); it also comprises genomes from North America (*n*=353), Southwest Indian Ocean Islands (*n*=184), East Asia (*n*=21), North Indian Ocean Islands (*n*=12), South Asia (*n*=12), Caribbean (*n*=12), Oceania (*n*=9) and Southeast Asia (*n*=8). Detailed information about the A1 genomes (*n*=1465) is given in Table S3.

**Fig. 1.**
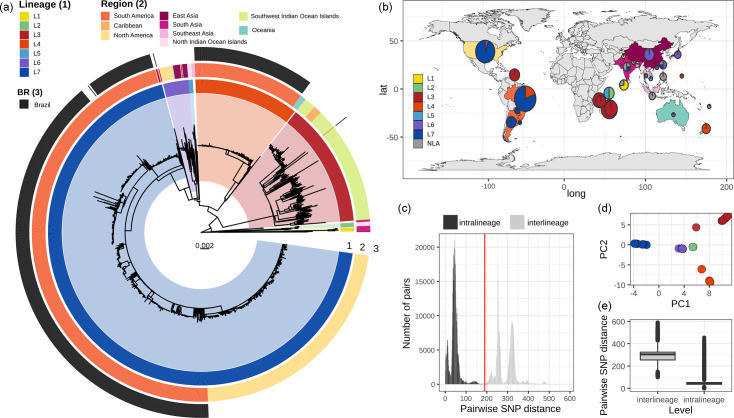
(a) Phylogenomic tree of 1465 genomes of *Xcc* pathotype A1. The assigned seven lineages (L1–7) are represented in the inner rings and highlighted in the tree. The second ring shows continent source information. Brazilian isolates are shown in black in the outer ring. (**b)** Comprehensive geographical distribution of the dataset visualized on the world map using filled circles coloured according to lineage frequencies.** (c)** Histogram of pairwise SNP distances between all genomes, coloured by lineage comparison. The red line marks 190 as an SNP distance cutoff used to define lineages. (**d)** PCA graphs representing the population structure information based on SNP alignment coloured according to lineage genotype. (**e)** Boxplots of pairwise SNP distances calculated within and between genomes belonging to each lineage.

The ML tree for the 1465 *Xcc* A1 genomes was constructed based on a core-genome alignment free of recombinant regions (Fig. 1a). To identify clusters in the phylogeny, we used both hierarchical Bayesian clustering with fastBAPS and core-genome SNP distance thresholds. The pairwise core-genome SNP distance distribution revealed peaks and troughs, which we used to set a threshold to define clades (Fig. 1c, e). A threshold of at least 190 SNPs separated 7 major clusters, concordant with monophyletic groups in the tree with 100% bootstrap support; these clusters were designated as lineages L1 through L7 (Fig. 1a). This distribution was also consistent with the structure observed in the PCA based on the SNP matrix (Fig. 1d). L7 includes most genomes from our dataset (*n*=1029, 70.3%), followed by L3 (*n*=197, 13.4%), L4 (*n*=165, 11.3%), L6 (*n*=40, 2.7%), L5 (*n*=7, 0.5%), L2 (*n*=7, 0.5%) and L1 (*n*=6, 0.4%). Fourteen (1%) other genomes were dispersed across the tree with distances greater than 200 SNPs from any other genome, and none were assigned to a lineage.

Although our dataset is not a systematic sampling across geographic regions, it can provide some rough insights into the global distribution of the A1 subclade (Figs 1b and S4). Some lineages show geographic structure. L3 contains isolates from the Southwest Indian Ocean Islands and the Caribbean Island Martinique (described in Richard *et al.* [24]). L4 is composed of isolates from South America (*n*=159) and Oceania (*n*=7). L6 is more widely dispersed, with isolates from North America (*n*=20), East Asia (*n*=18), Oceania (*n*=1) and Southeast Asia (*n*=1). L7 is mainly composed of isolates from the American continent (*n*=1027: South America, *n*=694, and North America, *n*=333) and 2 isolates from Asia (Fig. S4).

### *Xcc* A1 lineages diversified and acquired diverse gene sets in the process

The A1 pangenome resulted in 9973 genes, with a core genome (≥99% of all genomes) of 4129 genes. This core-genome size represents 94% of an average *Xcc* A1 genome (4395 coding sequences), showing that a majority of the genes encoded within a genome are conserved among all *Xcc* A1 genomes. The distribution of 1547 accessory genes (present in less than 99% of genomes but in at least 2 genomes) can be seen in a gene presence/absence matrix (Fig. S5A). We used PCA to illustrate the clustering based on the presence or absence of genes in the accessory genomes (Fig. S5B). Although most lineages have overlapping accessory genome content, 1022 out of 1029 L7 genomes were separated from other lineages (represented by the blue dots in Fig. S5B). Similarly, the majority of L4 genomes grouped together (159 out of 165). Principal component 2 shows the separation of 39 genomes belonging to the L7 and L3 lineages; we observed that all these genomes carry the pCuR plasmid (Table S4).

**Fig. 2.**
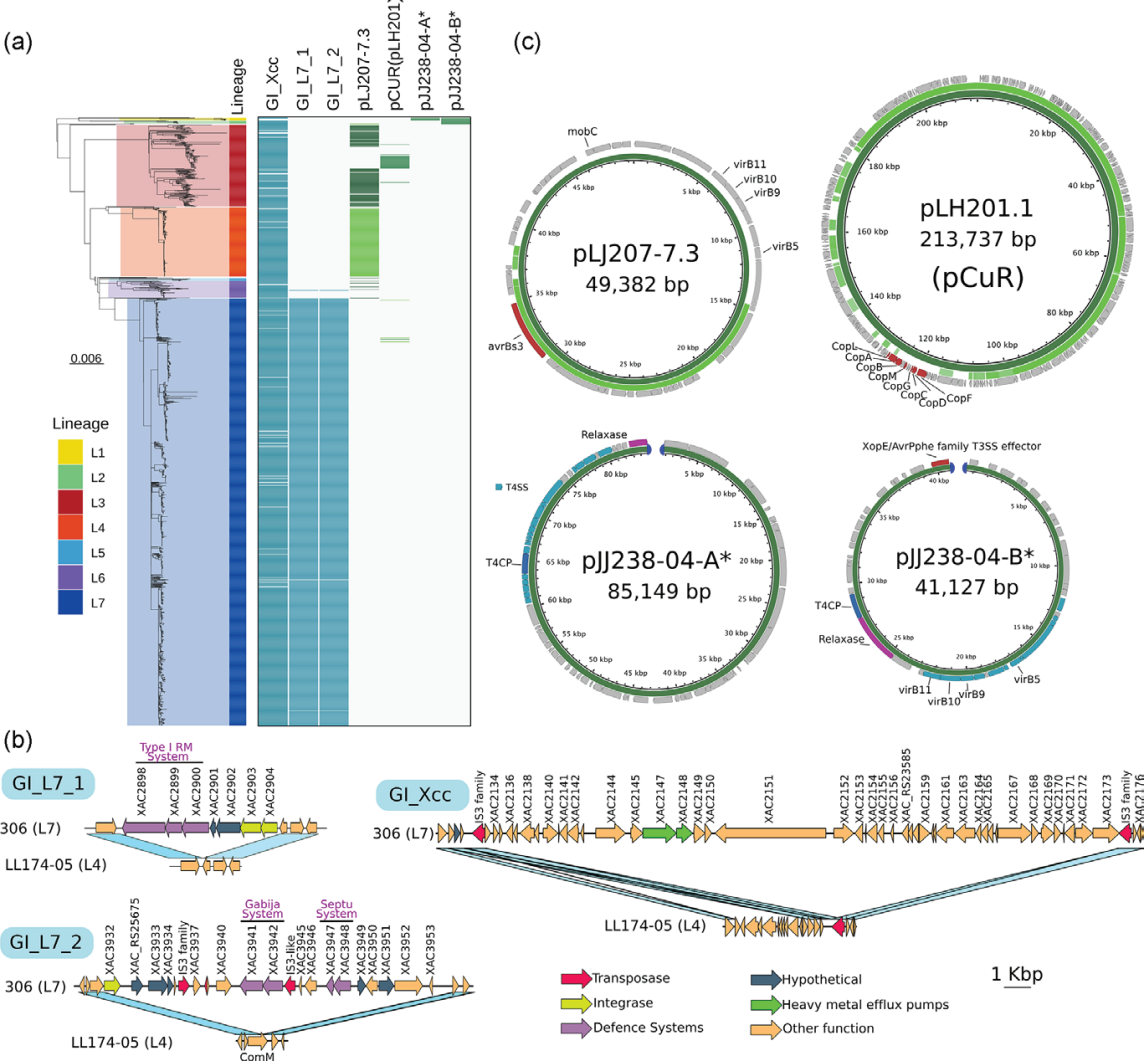
(a) Phylogenetic tree of *Xcc* A1 with lineage information in the first strip as shown in Fig. 1(a), alongside a presence/absence of GIs and plasmid sequences. Blue boxes represent GIs and green boxes indicate plasmids. For pLJ207-7.3 and pLH201.1 plasmids, the dark green boxes represent the full complete plasmids, whereas the lime green boxes represent the partial sequences of the plasmid. (**b)** Representation of GIs GI_L7_1, GI_L7_2 and GI_Xcc, with locus tags from reference strain A306. (**c)** Circle maps show the structure of variable plasmids in *Xcc* A1. The innermost dark green circle represents the full-length plasmid, whereas lime green regions represent the partial sequences that were observed in some genomes in our dataset. In pLH201.1, the cop operon is highlighted in red, as is the transcription activator-like effector gene in pLJ207-7.3 and pJJ238-04-B*. The plasmid sequence annotations are those from GenBank.

We identified several differentially present/absent genome regions between genomes from different lineages, including genomic islands (GIs) and plasmids (Fig. 2a). Two GIs of 14 and 29 kbp are present only in lineage L7, suggesting that its acquisition occurred in a common ancestor. GI_L7_1 is located in positions 3 398 500–3 413 000 in the reference A306 genome and contains 7 genes, including 3 genes of the type I restriction–modification (RM) system, integrases and hypothetical genes (Fig. 2b). GI_L7_2 is located in positions 4 622 000–4 651 000 and appears inserted in the middle of the *comM* gene sequence. This GI is composed of 23 genes and includes genes for the Gabija and Septu defence systems, insertion sequences, integrases and hypothetical proteins (Figs 2b and S6).

A plasmid pXac47-like (pLj207-7.3, 49.3 kbp), previously characterized in Richard *et al.* [47], is present in most L3 genomes (132 out of 197), as well as in 8 L6 and 1 L5 genomes. This plasmid carries a transcription activator-like effector (TALE) gene (AvrBs3-like). Additionally, a partial region of this plasmid (nearly 25 kbp) is carried by all L4 genomes and includes the TALE gene (Fig. 2c). Plasmid pLH201.1 (213.7 kbp) [47], carrying metal resistance-associated genes, is present in 35 genomes; 2 of these are in lineage L7 (both from Argentina) and 33 belong to lineage L3. This plasmid (also known as the pCuR plasmid) contains the operon *copLABMGCDF*, responsible for copper resistance, which has been observed in strains carrying this plasmid [48]. Additionally, we identified four genomes (three from Argentina and one from the USA) that have a contig similar to the pLH201 plasmid but lack the cop operon (Fig. 2c and Table S4).

Sequences identical to a linear contig from the *Xcc* JJ238-04 strain (GenBank accession JAABAX010000021.1) with 85 kbp were found in six genomes of the L1 lineage. This contig contains plasmid-associated genes (coding for a type 4 coupling protein (T4CP), a relaxase and a type IV secretion system gene cluster) but is not reported in the PLSDB database [49] (Fig. 2c). We also observed that sequences identical to another linear contig from the same strain (GenBank accession JAABAX010000067.1) with 41.1 kbp are present in 16 genomes belonging to the L1 and L2 lineages. This contig also contains a T4CP gene, a relaxase gene and a T4SS gene cluster, in addition to a TALE gene (Fig. 2c). The presence of T4CP and T4SS genes suggests that this sequence corresponds to a plasmid; a similar plasmid (nt identity ~99%) was previously described in *Xcc* pathotype A* in Iran (GenBank accession: JRON01000182.1).

Interestingly, 54 genomes lacking a chromosomal region of around 60 kbp (named GI_Xcc in Fig. 2b) were scattered throughout the phylogeny. This highly polymorphic region corresponds, in the A306 reference genome, to positions 2 488 375–2 548 769, which contains 42 genes and is flanked by IS3 sequences. We note that genes *czc*A (XAC2147) and *czc*B (XAC2145) found in this region code for heavy metal pumps that have been associated with resistance to various metals including cadmium, zinc and cobalt in *Alcaligenes eutrophus* [50] (Fig. 2b).

### The vast majority of genomes from Brazil belong to just two lineages

**Fig. 3.**
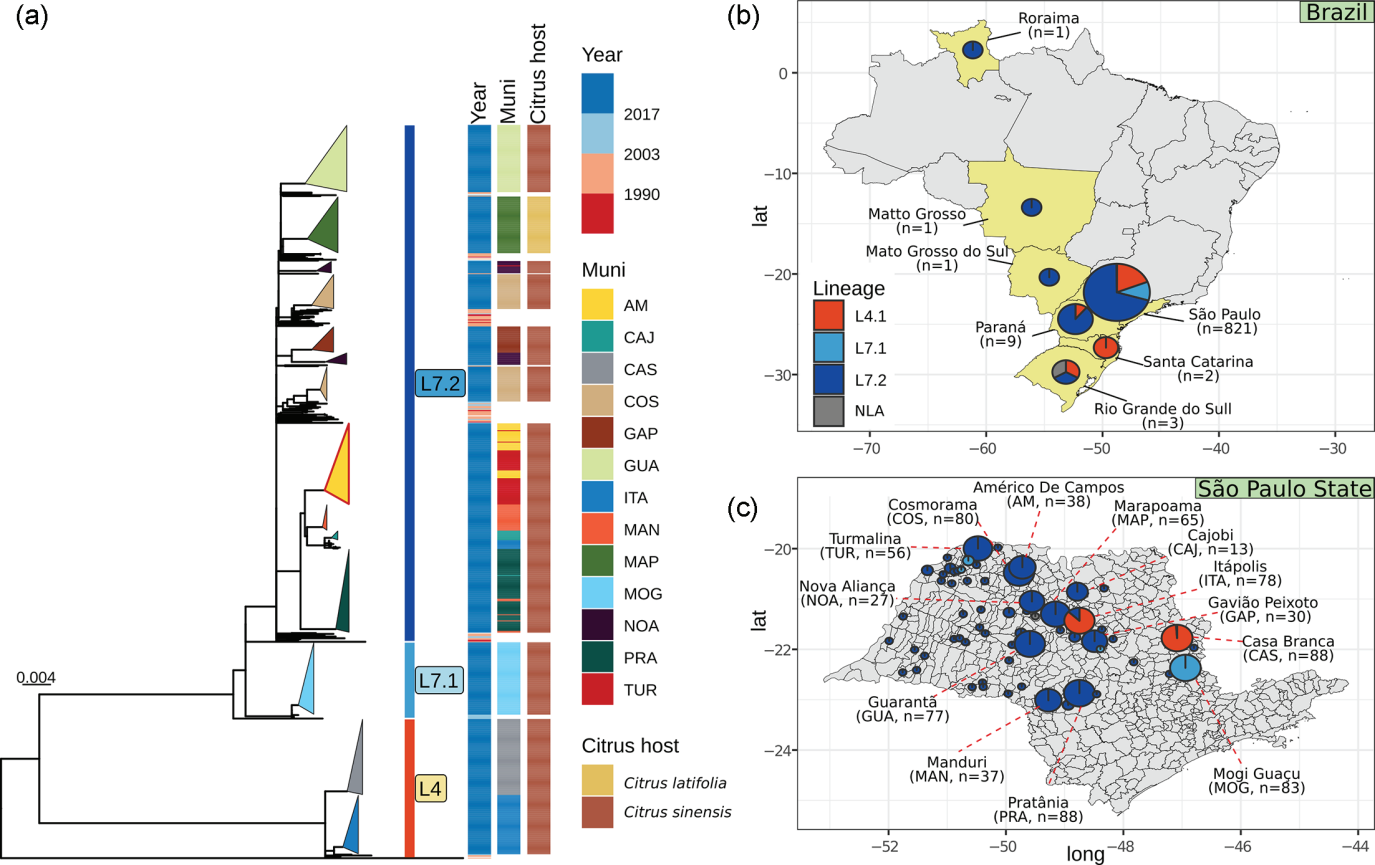
(a) Phylogenetic tree of 841 *Xcc* A1 Brazilian genomes including information on subclade assignment and year and location of isolation.** (b)** Geographical distribution of 841 Brazilian isolates. (**c)** Geographical distribution of 821 *Xcc* A1 genomes in SP state by municipality.

The Brazilian dataset (*n*=841) includes 83 historical (isolated in the period 1976–2014) and 758 contemporary genomes (isolated in the period 2018–2021). Most isolates were sampled in SP state (*n*=821). The contemporary isolates were sampled across 13 municipalities of SP state (Table S5). The number of isolates ranged from 13 to 88 genomes per municipality. Of the 83 historical isolates, 63 were sampled from 53 different municipalities within SP state (Fig. 3c) and the rest (*n*=21) from the following other states: Paraná (*n*=9), Rio Grande do Sul (*n*=3), Santa Catarina (*n*=2), Mato Grosso do Sul (*n*=1), Roraima (*n*=1) and Mato Grosso (*n*=1). The isolation locations for three isolates are unknown.

As observed in the global phylogeny (Fig. 1a), the Brazilian isolates are distributed among the L7 (*n*=682), L4 (*n*=159) and L3 (*n*=1) lineages. We generated a phylogeny using only the Brazilian isolates and were able to observe that L7 subdivides into two subclades: L7.1 and L7.2 (Fig. 3a). The median distance between these subclades was 64 SNPs (interquartile range 61–68). The median distance between isolates within the L7.2 subclade was 44 SNPs (interquartile range 36–49). The median distance between pairs of L7.1 genomes was five SNPs (interquartile range 4–7), strongly indicative of recent clonal expansion (Fig. S7).

Isolates belonging to the L7.1 subclade were only found in SP state. The L7.2 subclade was identified in nearly all sampled states, except Santa Catarina, where the two isolates observed belong to lineage L4 (Fig. 3b). The L7.2 lineage was widespread across the SP citrus belt (Fig. 3c), whereas L7.1 isolates were sampled in the Mogi Guaçu municipality (*n*=83) and Palmira d’Oeste (*n*=1), Urania (*n*=2) and Boa Esperança do Sul (*n*=1). In SP state, the contemporary L4 isolates were found only in municipalities Casa Branca (*n*=68) and Itápolis (ITA) (*n*=87).

Generally, contemporary isolates from the same municipality (in lineages L7.1, L7.2 and L4) grouped in the tree, separate from other municipalities, and were genetically almost identical, with an average SNP distance of only ten SNPs, suggesting a clonal expansion as already mentioned (Fig. S7). The exceptions were the genomes from Américo de Campos and Turmalina, which grouped in a single cluster. Both municipalities are located in the mesoregion São José do Rio Preto. On the other hand, in some municipalities, the isolates were distributed in two different clusters, as observed for Cosmorama, Nova Aliança and ITA (Fig. 3a).

### Temporal evolution and spread of *Xcc* A1 in Brazil

Temporal phylogenetic inference of the Brazilian *Xcc* A1 population was obtained from a representative dataset of 203 genomes, which included 72 historical genomes and 131 contemporary genomes with available collection dates (Table S6). The latter 131 genomes were subsampled from all 758 contemporary genomes, considering the municipality and clade position in the phylogeny in Fig. 3(c) and choosing at random when having more than 1 genome per municipality or clade position.

**Fig. 4.**
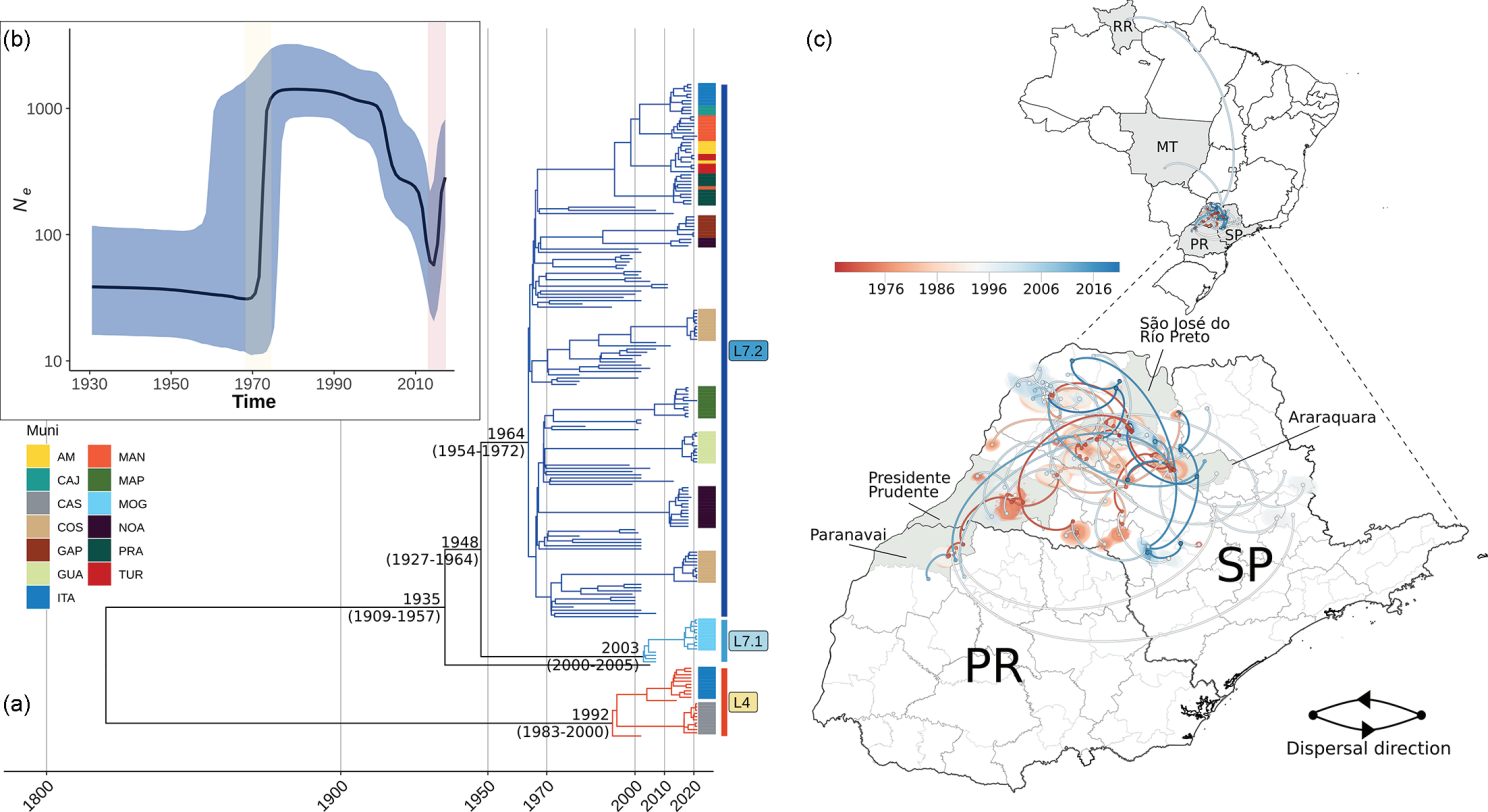
(a) Maximum clade credibility tree produced using BEAST (lognormal relaxed clock model; Bayesian skyline) of a subsample of 203 Brazilian isolates.** (b)** Bayesian skyline plot showing temporal changes since 1930 in effective population size (black curve), with 95% confidence intervals (blue shading). Vertical shade areas highlight the rapid-grow phase around 1970 (yellow shade) and after 2010 (red shade). (**c)** Continuous phylogeographic reconstruction of the spread of the L7.2 lineage in Brazil and in (zoomed in part) SP and Paraná (PR) states. Circles represent internal nodes of the phylogeny and are coloured according to their inferred time of occurrence. Shaded areas represent the 80% highest posterior density interval and depict the uncertainty of the phylogeographic estimates for each node. Solid curved lines denote the links between nodes and dispersal direction (counterclockwise).

According to this reconstruction (Fig. 4a), the emergence of the L7 lineage occurred in 1935 (95% Highest Posterior Density (HPD): 1909–1957). An internal node is dated to 1948 (95% HPD: 1927–1964). The most recent common ancestor (MRCA) of the L7.2 clade emerged around 1964 (95% HPD: 1954–1972); for L7.1, the emergence estimate is 2003 (95% HPD: 2000–2005), and for L4, it is 1992 (95% HPD: 1983–2000). We estimated a mean substitution rate of 1.425×10^−7^ per site per year (95% HPD: 9.274×10^−8^–1.871×10^−7^).

The effective population size trajectories were estimated using the Bayesian skyline demography model in BEAST v3.10.4 (Fig. 4b), with the aim of determining the evolutionary dynamics of Brazilian isolates. The skyline plot shows a swift expansion from the early 1970s, sustained until 2000, followed by a subsequent decline and resurgence in 2010.

We performed a phylogeographic analysis of the L7.2 lineage (168 isolates with available collection dates and locations) (Fig. 4c, Table S6). Our study suggests that L7.2 emerged in the São Jose do Rio Preto microregion and spread to Araraquara, Presidente Prudente and Paranavaí (Paraná state), in the early 1970s. According to this analysis, after the initial surge, other novel strains started to occur over a 40-year period throughout the SP citrus belt (coloured circles in Fig. 4c).

### Positive selection on SodM linked to ongoing selective pressure

**Fig. 5.**
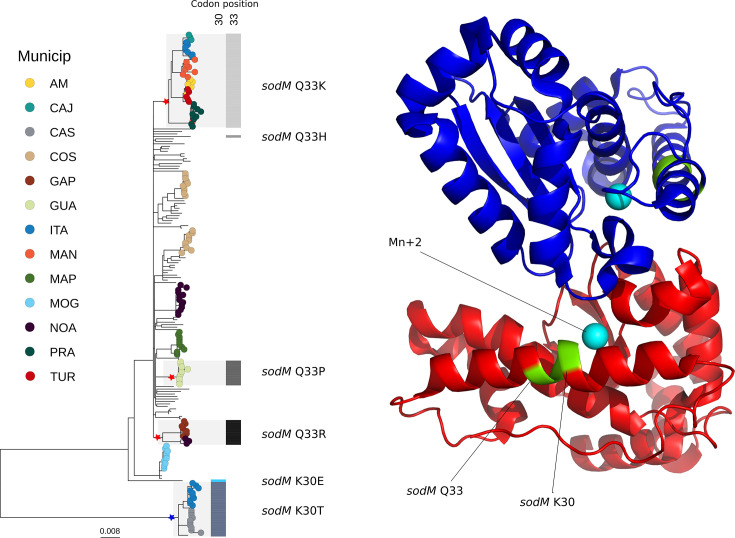
The ML tree of 203 Brazilian isolates, highlighting inferred mutational events for SodM. Each star marks the position where mutations accumulated. Tip points are coloured in contemporary isolates from 13 SP municipalities. The right panel shows a 3D representation of SodM protein dimer (XAC2386) based on the PDB entry 6BEJ. The polymorphic residues K30 and Q33 are indicated in green, and the cyan spheres represent the Mn+2 ion.

Given the temporal and geographical specificity of the Brazilian *Xcc* A1 population, we decided to check for signs of evolutionary selection in the genes of these genomes. Using the representative dataset of 203 genomes mentioned previously, we screened for positive selection patterns using FUBAR in each gene alignment. The test identified five genes that underwent positive selection (Table 1). Interestingly, one gene showed temporal signs of selection with variation observed in contemporary isolates. The superoxide dismutase gene (*sodM*, locus tag: XAC2386) showed non-synonym variation in positions 30 and 33 of the protein. A total of six unique non-synonymous mutations in Brazilian isolates were detected (K30T, K30E, Q33K, Q33R, Q33H and Q33P) (Fig. 5).

**Table 1.** List of genes under positive selection identified by FUBAR

Locus tag	Name	Function	Start	End
XAC0593	–	GGDEF domain-containing protein	696,494	698,368
XAC_RS26625	–	Hypothetical protein	699,564	699,722
XAC1880	*rpfB*	Long-chain fatty acid – CoA ligase	2,182,586	2,184,268
XAC2386	*sodM*	Superoxidase dismutase	2,780,758	2,781,369
XAC3001	–	Major Facilitator Superfamily (MFS) transporter	3,512,473	3,513,846

Start and end positions refer to the gene locus tag in reference genome A306.

SodM is a metalloprotein involved in the oxidative stress response and is essential for the viability of *Xcc* [51,52]. SodM positions 30 and 33 belong to the alpha1 helix [44]. These residues are in the neighbourhood of aas that interact around the active site and aid in securing the substrate superoxide anion (His27, His31 and Tyr35). An *in silico* assessment of the impact of mutations on the protein structure was performed using DDMut software [43]. K30T and Q33P were predicted to be destabilizing mutations, whereas Q33K and Q33R were predicted to be stabilizing mutations (Table 2). Our assessment suggests that these mutations generate little impact on structure, but functional analysis is necessary to evaluate the impact on its enzymatic activity.

**Table 2.** Prediction of protein stability changes in SodM mutations using DDMut

	Mutation	Predicted stability change (ΔΔGStability wt->mt)	Predicted effect
1	K30T	−0.48 kcal/mol	Destabilizing
**2**	Q33P	−0.9 kcal/mol	Destabilizing
**3**	Q33K	0.23 kcal/mol	Stabilizing
**4**	Q33R	0.18 kcal/mol	Stabilizing

### Recombining regions

A final investigation into the specificities of the genomes belonging to the L7 lineage was made with the Gubbins tool, which predicts recombining regions. The results are shown in Table S7. An IS3 family transposase was found flanking recombinant region 2 (positions 2 834 411–2 839 664 in reference strain A306), downstream at positions 2 830 363–2 831 588. Recombinant region 3 (positions 3 799 550–3 805 347) contains a Tn3 gene and effector genes of the type III secretion system (*XopAI* and *AvrXacE2*). An IS3 family transposase pseudogene was identified downstream at positions 3 795 904–3 797 071, and an IS1595 family transposase was found upstream, at positions 3 808 805–3 809 920.

## Discussion

A large and diverse genome collection enhances our capacity to investigate pathogen emergence and dissemination [53,54]. *Xanthomonas* species have been the focus of some recent large genome surveys [55,57]. In this study, we report 758 new *Xcc* A1 genomes from SP state in Brazil and use a population genomic approach to study the *Xcc* A1 global population diversity and reconstruct the evolutionary history from its introduction into Brazil to the present. The number of genomes sequenced for this work far surpasses those of previous *Xanthomonas* studies cited earlier.

We used a pairwise SNP divergence approach to identify robust lineages that give structure to the global diversity of *Xcc* A1. SNP-based genotyping frameworks have been previously adopted for lineage classification in other bacterial pathogens, such as *Shigella sonnei* [58] and *Mycobacterium tuberculosis* [59]. This approach facilitates the identification and communication of epidemiologically important lineages from genome data, which may be important for surveillance purposes. Using this approach, we show that the majority of *Xcc* A1 isolates are distributed into seven major lineages.

Although accessory gene presence/absence was not concordant with lineage classification, differential presence of GIs and plasmids was observed across the phylogeny. We interpret that the L7 lineage emerged by acquiring two GIs carrying three distinct defence systems, including one type I RM system. Defence systems are key players in the defence against phages or other invasive DNA elements [46]. In several bacterial species, the differential repertoire of RM systems shapes the diversification and pathogenicity of populations by discriminating mobile genetic elements (MGEs) [60,62]. Additionally, we found that *comM*, which encodes for a helicase involved in homologous recombination [63], was disrupted by GI_L7_2. This gene is often inactivated by the integration of diverse MGEs in several species, including *Acinetobacter baumannii* [64]. The inactivation of this gene may reduce the transformation rate, impacting MGE mobilization [65]. We hypothesize that the gain of defence systems and inactivation of *comM* may reduce the capacity of L7 isolates to acquire plasmids and other elements.

Horizontal acquisition of genes or genomic regions providing functional advantage may favour the spread and adaptability of pathogenic bacteria [66]. For example, the acquisition of gene clusters coding for type III-secreted proteins has been associated with the emergence and dissemination of *Xanthomonas vasicola* in the USA and Argentina [67]. Plasmids are important in *Xanthomonas* biology since they code for genes relevant in host–pathogen interactions, such as secretion systems, effectors or metal resistance operons [68,69]. The pLJ207-7.3 plasmid carrying a TALE gene was identified in the Southwest Indian Ocean Islands clade (L3 lineage) [70], and we found a partial sequence of this plasmid in the L4 lineage. Plasmid rearrangement in *Xanthomonas citri* has been characterized using long-read sequencing [71]. Due to the limitations of short-read sequencing, we cannot determine whether the partial pLJ207-7.3 sequence in the L4 lineage forms a chimeric plasmid with other plasmids (such as pXac33 or pXac64) or whether it represents a novel plasmid itself.

Our results show that the vast majority of genomes from Brazil were included in only two lineages, L4 and L7, which allowed us to conduct a temporal evolutionary analysis and correlate the results with the emergence of citrus cultivation and disease in Brazil. Citrus canker was first reported in South America (in Brazil) in 1957 [8]. We dated the emergence of the L7 lineage to 1935 (95% HPD: 1909–1957) (Fig. 4a). Although the 95% HDP range includes the date of the first reported case, it is possible that there were multiple introductions of this lineage in Brazil. For example, one internal node was dated to 1948 (95% HPD: 1927–1964), which is in better agreement with the epidemiological information. The substitution rate was inferred in 1.425×10^−7^ substitutions per site per year, and a similar value (1.43×10^−7^) was obtained previously for *Xcc* [45].

Our results also show that L7 can be subdivided into two subclades (L7.1 and L7.2), allowing a more refined temporal analysis. The L7.2 subclade expanded throughout Brazil and SP shortly after its estimated emergence in 1964 (95% HPD: 1954–1962). On the other hand, L7.1 was only identified in SP, specifically in Mogi Guaçu, with an MRCA of L7.1 emerging at the beginning of the 2000s [2003 (95% HPD: 2000–2005)], suggesting that it may have been imported from another country. Possible sources might be Argentina and Florida, since isolates from these places are at the base of this clade. We remark that the Mogi Guaçu municipality is one of the regions where citrus canker has been detected most recently in SP state [72].

More than one lineage can coexist in the same region or country. In addition to the L7 lineage, the second most prevalent lineage in Brazil is L4, which has been detected in SP state as well as in other states. The emergence of the L4 Brazilian lineage was estimated to be in 1992 (95% HPD: 1983–2000). The closest relatives of this clade were from Oceania (New Zealand and Guam).

According to our inference, the L7 sublineage diversification occurred approximately at the same time as the rise in the estimated effective *Xcc* A1 population (Fig. 4b), around the year 1970. This may be linked to the expansion of the orange juice industry in Brazil. The SP state orange juice industry emerged in the 1960s as a response to decreased production in Florida caused by frequent freezes and rising demand in the US market [73]. These circumstances fuelled a rapid expansion in orange juice production, with exports surging from 5313 MT in 1963–1964 to nearly 1.3 million MT by 1999–2000 [74].

Our results also indicate a period during which the effective population of *Xcc* A1 declined between 2000 and 2010 (Fig. 4b). This decline coincided with a time when the eradication protocol for canker became more stringent in infected orchards (total removal of citrus blocks when disease incidence exceeded 0.5%) [9]. Despite these efforts, changes in the eradication protocol in SP state in 2009 contributed to an increase in citrus canker, rising from 0.14% of contaminated blocks in 2009 to ~1% in 2011 [75]. This coincides with a subsequent rise and sublineage diversification of XccA lineages starting from 2010 to the present.

In spite of the more recent emergence of sublineage 7.1, we also note that sublineage 7.2 still appears to be the dominant lineage in the SP citrus belt, being present in 59 out of 65 sampled municipalities (L7.1 was detected in only 4 municipalities). This suggests that the eradication programmes had little or no effect on the genomic makeup of the presumptive dominant lineage over a time span of ~50 years. This observation is consistent with the fact that such programmes have for the most part targeted the host plants, and not the pathogen.

Our geographic-temporal analysis of the 7.2 sublineage (Fig. 4c) is also in agreement with what has been reported in the literature with respect to the emergence of citrus canker in the SP – Paraná citrus belt. As mentioned in the Introduction, the first reported emergence of citrus canker was in the Presidente Prudente region [8], which is among the red-coloured regions (and hence earlier in time) in Fig. 4(c). In the 1980s and 1990s, citrus canker moved northwards [9,76]. And indeed, nearly all blue-coloured regions (later in time) in Fig. 4(c) are to the north of the south-coloured regions.

It has been suggested that positive selection is the main force of evolution in *Xcc* [12]. We identified five genes under positive selection in our dataset. From these, *sodM* showed temporal variation. SodM plays an important role during bacterial pathogenesis, likely involved in defence against reactive oxygen species, which plants commonly produce to combat pathogens during infection and colonization [52]. Recent work has suggested that *sodM* also contributes to alleviating toxicity produced by heavy metals including Cu^+2^ in several bacteria species [77]. Evidence of this is the induction of Mn-SOD expression when bacteria is exposed to excess metal [77]. Elevated levels and activity of SOD would safeguard metal-exposed cells by reducing superoxide concentrations. The high prevalence of mutations in *sodM* across the tree suggests that they may be adaptive. Functional experiments on the enzymatic activity of this protein will be necessary to evaluate the effect of these mutations.

In conclusion, our results present a detailed picture of the history and current status of *Xcc* A1 strains in Brazil, especially in the SP citrus belt. The phylogeographic analysis results are in agreement with field observations for the past 50 years and reinforce the notion that *Xcc* A1 is an endemic species that has been little or not affected by eradication programmes. In 2017, a law was passed in SP state allowing copper sprays in affected orchards. Such practices may have an effect on citrus canker that may sharply differ from what has been seen so far. The analyses presented here should be able to detect such effects, if the necessary strain DNA sequencing is carried out. This should be the target for researchers, farmers and policy makers concerned about the Brazilian citrus industry in the near future.

## supplementary material

10.1099/mgen.0.001338 Supplementary Material 1.

10.1099/mgen.0.001338 Uncited Supplementary Material 2.

## Abbreviations

CCEP: citrus canker eradication programme
GI: genomic island
GTR: general time reversible
MCMC: Markov chain Monte Carlo
MGE: mobile genetic element
ML: maximum likelihood
MRCA: most recent common ancestor
PCA: principal component analysis
RM: restriction–modification
SP: São Paulo
TALE: transcription activator-like effector
T4CP: type 4 coupling protein
*Xcc*: *Xanthomonas citri* subsp. *citri*
