# Supplementary material for: Evolution and spread of Xanthomonas citri subsp. citri in the São Paulo, Brazil, citrus belt inferred from 758 novel genomes
Source: Microb Genom. 2025 Jan 16;11(1):001338. doi: 10.1099/mgen.0.001338 (PMC11736806; doi:10.1099/mgen.0.001338)
Supplement: Supplementary Material 1. [file mgen-11-01338-s001.pdf]

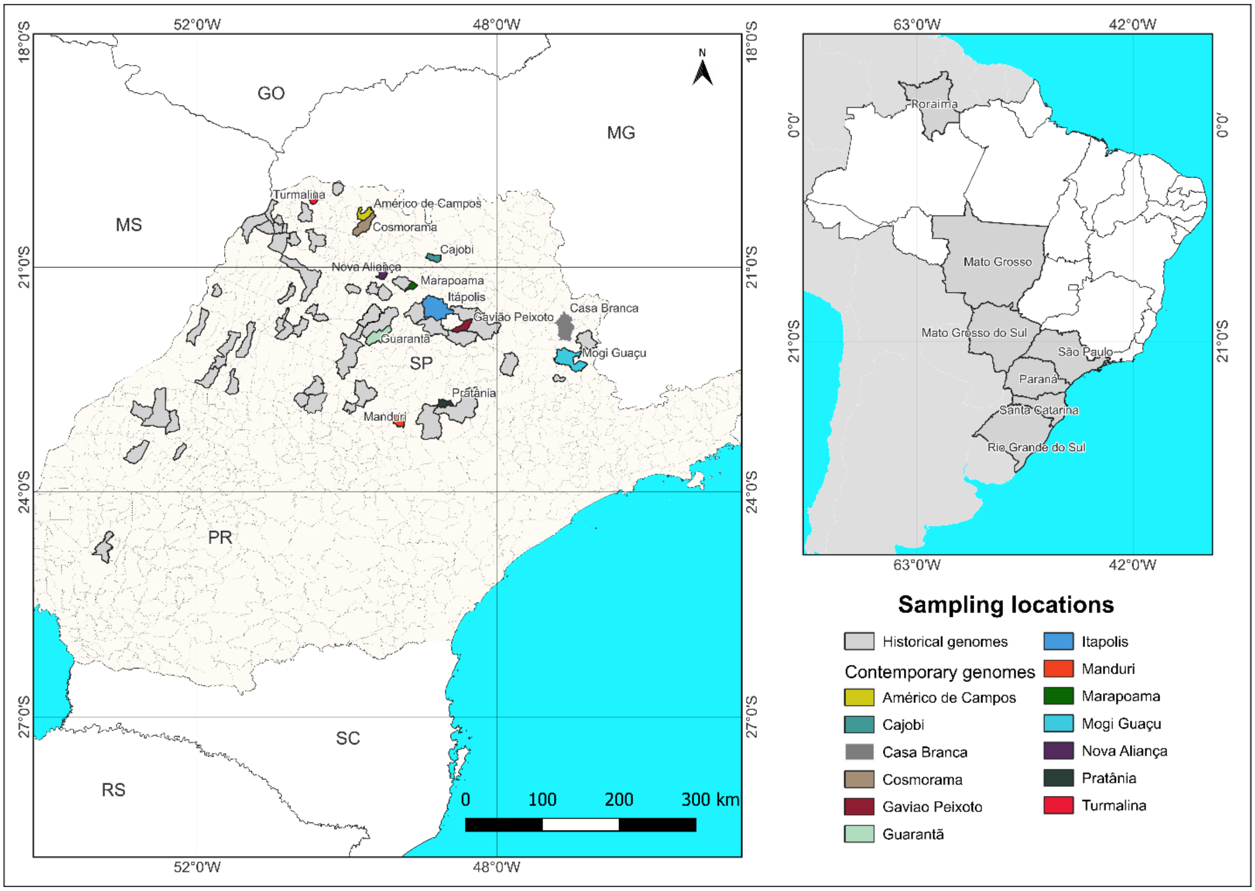


**Figure S1.** Sampling locations for isolates in Brazil (*n*=841). Brazilian states colored in gray (rightmost map) give the provenance of historical isolates (*n*=83) from São Paulo (*n*=63), Paraná (*n*=9), Rio Grande do Sul (*n*=3), Santa Catarina (*n*=2), Mato Grosso do Sul (*n*=1), Roraima (*n*=1), Mato Grosso (*n*=1) and unknown locations (*n*=3). In the detailed map (to the left), gray areas give the provenance of historical isolates in terms of municipalities in São Paulo (SP) and Paraná (PR) states; colored areas represent the municipalities where contemporary isolates were sampled (*n*=758).

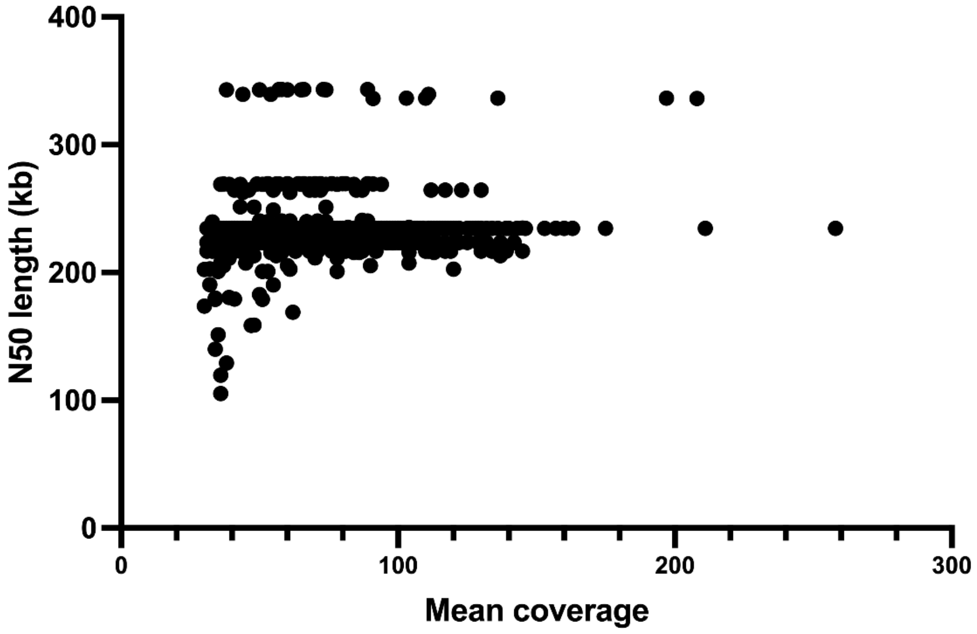


**Figure S2**. Scatterplot showing assembly coverage versus N50 length (in kbp) for the 758 XccA genomes sequenced for the present work.

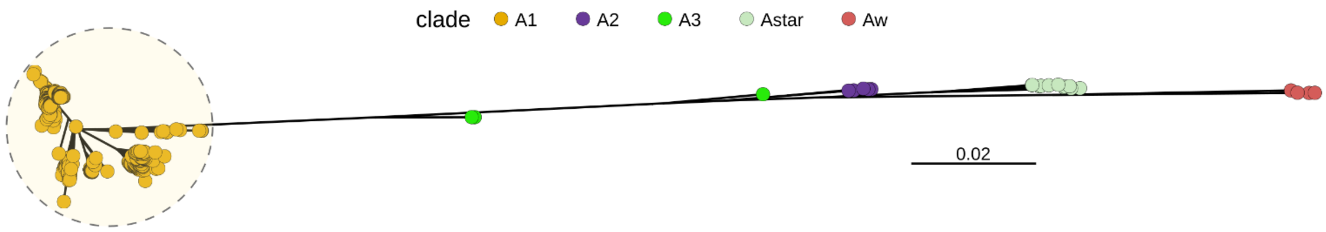


**Figure S3**. Unrooted maximum likelihood phylogeny of *Xanthomonas citri* subsp. *citri*.

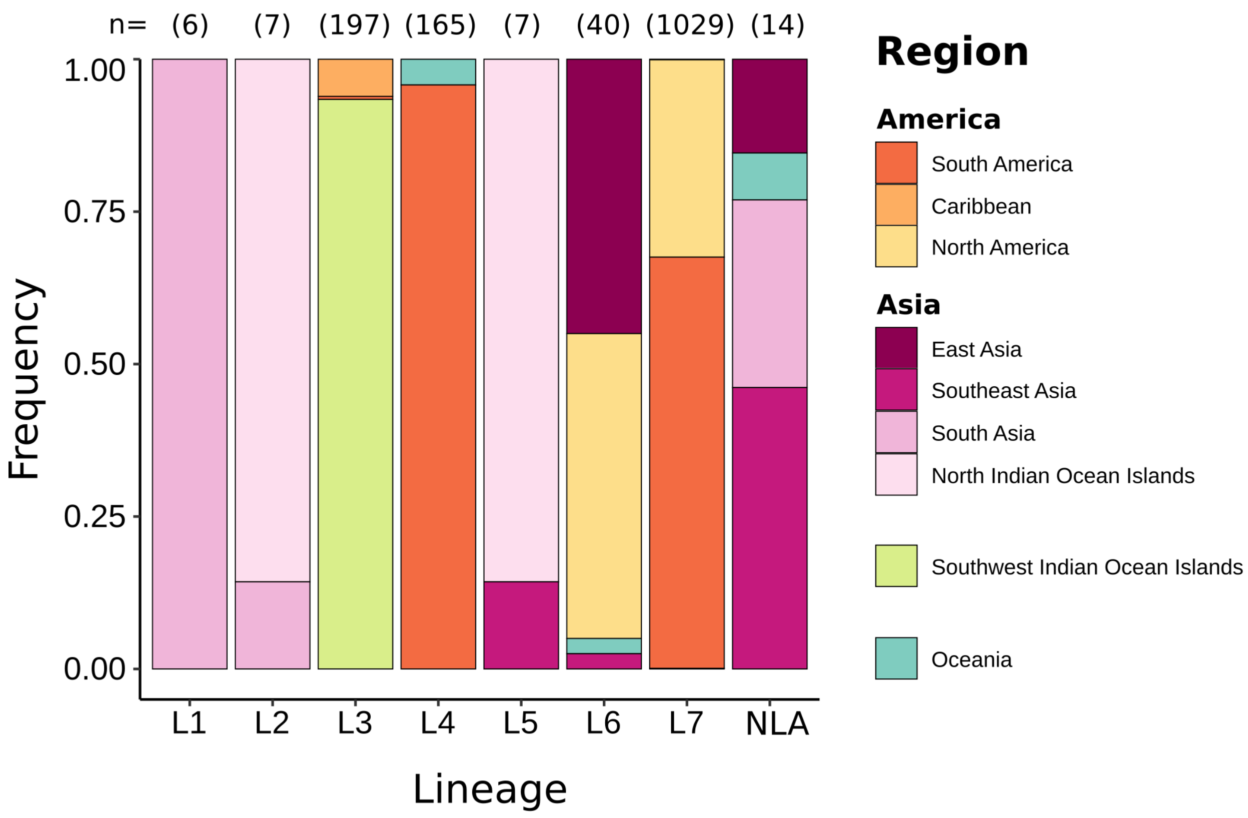


**Figure S4**. Frequencies by lineage for all genomes and colored by region of isolation. On top of each colored bar we show the number of genomes classified in that lineage.

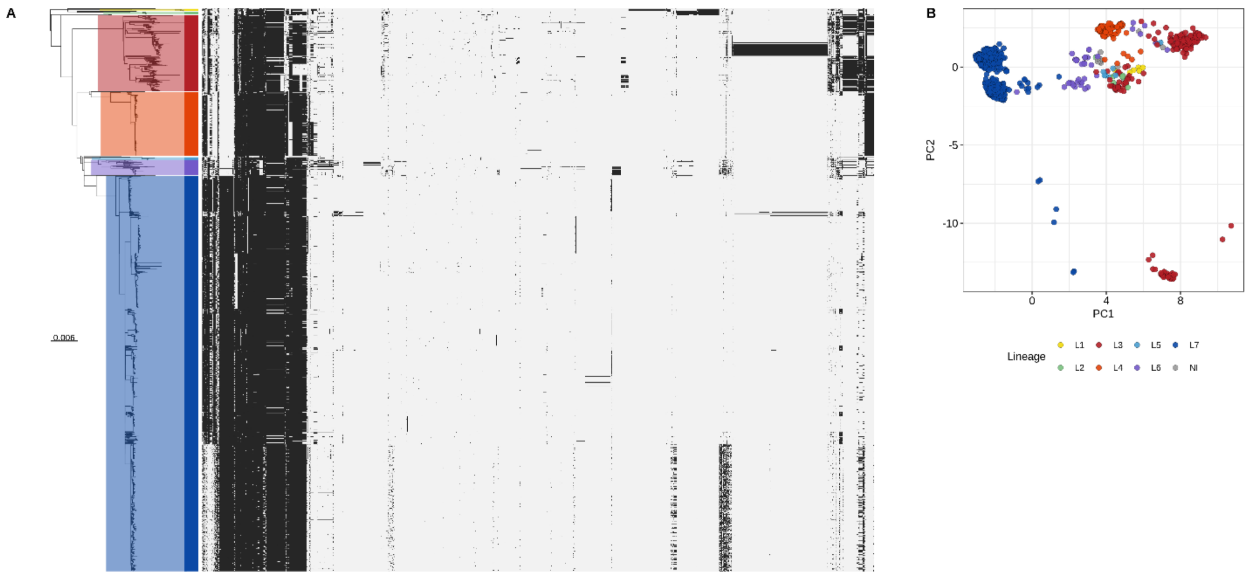


**Figure S5. A.** Phylogenetics tree of 1465 Xcc A1 genomes alongside the presence/absence patterns of 1,547 accessory genes, with gene presence indicated by black bars and gene absence by white bars. **B.** PCA based on the presence/absence accessory matrix. Circles represent genomes and colors represent lineages.

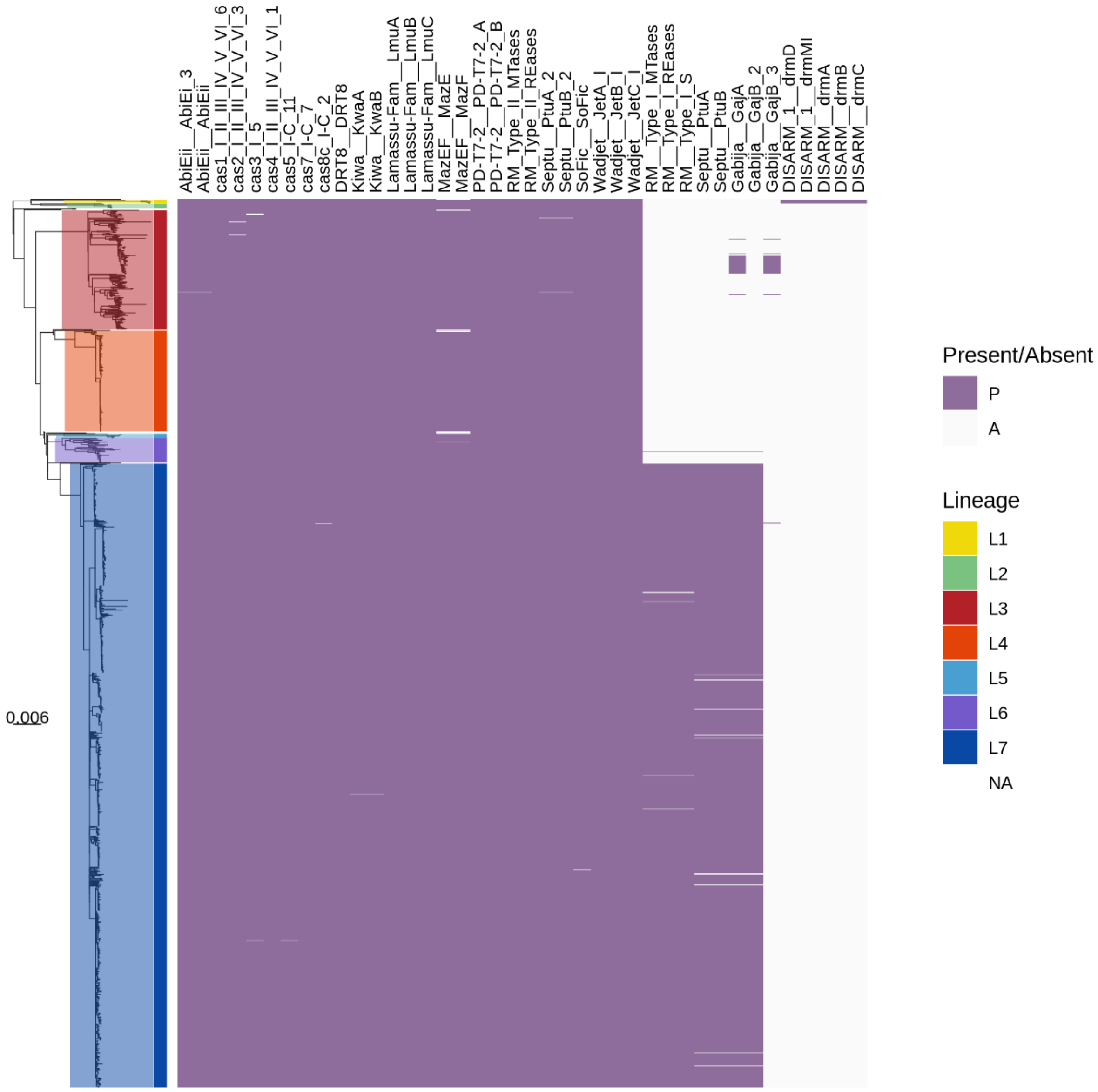


**Figure S6.** Heatmap showing presence/absence of bacteriophage defense systems in the 1,465 XccA genomes analyzed in this study, as predicted by program DefenseFinder. The defense systems are shown on top of each column. The XccA clades are colored according to the lineages described in the main text.

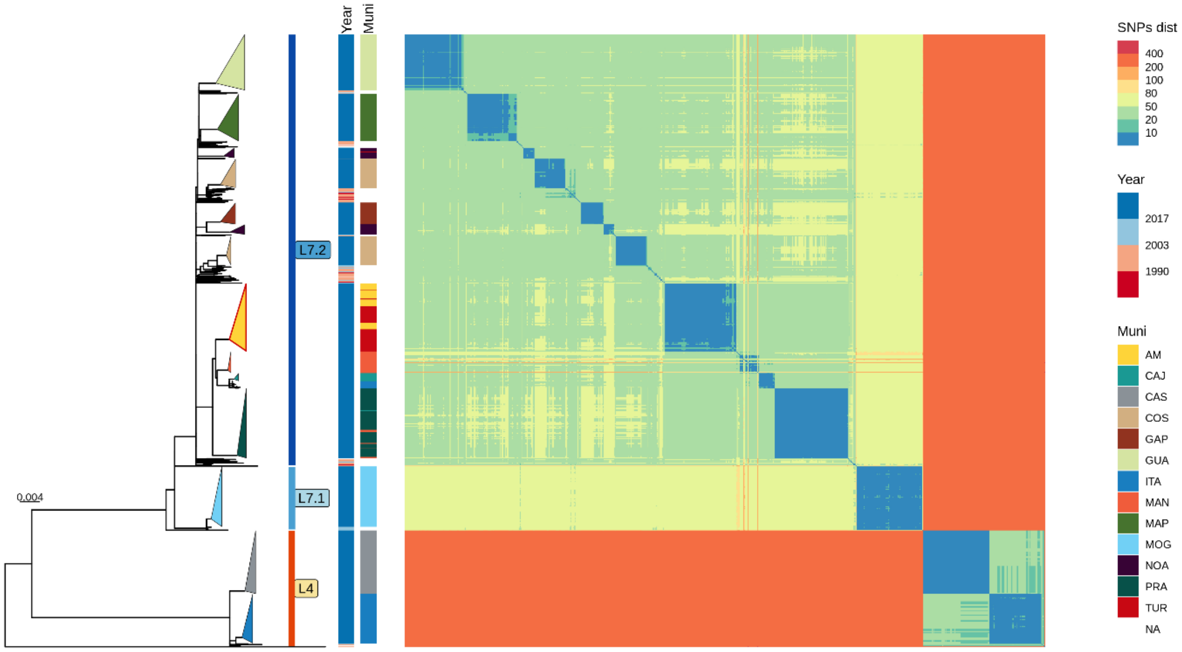


**Figure S7. S**NP distance matrix between 841 *X. citri* subsp. *citri* Brazilian isolates.
